# Influenza virus infection enhances tumour-specific CD8^+^ T-cell immunity, facilitating tumour control

**DOI:** 10.1371/journal.ppat.1011982

**Published:** 2024-01-25

**Authors:** Philine Steinbach, Eva Pastille, Lara Kaumanns, Alexandra Adamczyk, Kathrin Sutter, Wiebke Hansen, Ulf Dittmer, Jan Buer, Astrid M. Westendorf, Torben Knuschke

**Affiliations:** 1 Institute of Medical Microbiology, University Hospital Essen, University of Duisburg-Essen, Essen, Germany; 2 Institute for Virology, University Hospital Essen, University of Duisburg-Essen, Essen, Germany; Icahn School of Medicine at Mount Sinai, UNITED STATES

## Abstract

Influenza A virus (IAV) can cause severe respiratory infection leading to significant global morbidity and mortality through seasonal epidemics. Likewise, the constantly increasing number of cancer diseases is a growing problem. Nevertheless, the understanding of the mutual interactions of the immune responses between cancer and infection is still very vague. Therefore, it is important to understand the immunological cross talk between cancer and IAV infection. In several preclinical mouse models of cancer, including melanoma and colorectal cancer, we observed that IAV infection in the lung significantly decreased the tumour burden. Concomitantly, tumour-specific CD8^+^ T-cells are strongly activated upon infection, both in the tumour tissue and in the lung. CD8^+^ T-cell depletion during infection reverses the reduced tumour growth. Interestingly, IAV infection orchestrated the migration of tumour-specific CD8^+^ T-cells from the tumour into the infected lung. Blocking the migration of CD8^+^ T-cells prevented the anti-tumoural effect. Thus, our findings show that viral respiratory infection has significant impact on the anti-tumour CD8^+^ T-cell response, which will significantly improve our understanding of the immunological cross talk between cancer and infection.

## Author summary

The Influenza A virus (IAV) causes severe respiratory infections during seasonal outbreaks, while the rising incidence of cancer presents a growing health concern, yet our understanding of the interactions between immune responses to infection and cancer is still unclear. Therefore, we aimed to decipher the immunological cross talk between cancer and IAV infection.

Using preclinical mouse models of melanoma and colorectal cancer, we made intriguing observations. IAV infection in the lung led to a significant reduction in tumour growth. Additionally, the infection activated tumour-specific CD8^+^ T-cells, crucial components of the immune system’s cancer-fighting ability, both in the lung and tumour tissue. Remarkably, when CD8^+^ T-cells were depleted during infection, the tumour growth reduction was reversed.

It appears that IAV infection orchestrates the migration of tumour-specific CD8^+^ T cells from the tumour into the infected lung, since blocking migration abolished the anti-tumour effect. These findings shed light on the significant impact of viral respiratory infection on the anti-tumour CD8^+^ T-cell response, advancing our understanding of the intricate interplay between cancer and infection. Such insights hold promise for tackling these complex health challenges more effectively.

## Introduction

Influenza is a serious mucosal respiratory disease that regularly becomes a global health problem through seasonal epidemics [1]. Therefore, as tumour incidence has steadily increased worldwide over the past few decades, it is important to understand the impact of such infection on other diseases such as cancer. Although cancer therapy options evolved, many melanoma patients still do not respond to the offered therapies [2,3]. In this regard, an increasing focus lies on targeting the tumour microenvironment (TME). Immune cells are an important part of the TME. Here, the role of immune cells in the TME can be either to suppress tumour formation or to promote tumorigenesis. The presence of cytotoxic CD8^+^ T-cells in the TME is often associated with a positive prognosis in cancer patients [4–6]. On the other hand, cells with an immunosuppressive effector phenotype, such as regulatory T-cells (Tregs) or the immunosuppressive lineage of macrophages, can promote tumour development and progression by dampening the anti-tumoural immune response [7,8]. In general, cancer-associated fibroblasts also promote an immunosuppressive phenotype through the production of immunomodulatory chemokines and cytokines [9]. This highlights that the TME can have a strong inhibitory effect on anti-tumour immune responses, thus significantly influencing the course of the tumour disease. one reason for failure of therapy is the lack of infiltration of effector T-cells or rapid dysfunction of T-cells due to exhaustion by inhibitory signals [10]. Therefore, reactivation of cancer-fighting immune cells such as tumour-infiltrating cytotoxic CD8^+^ T-cells during tumorigenesis is of great interest. Importantly, infections and cancer diseases are two of the most common severe human diseases, so that an interaction of the respective immune responses is obvious. However, very little information is available on the consequences of concomitant non-oncogenic infection and cancer. Recent studies describe that pathogen-specific CD8^+^ T-cells support the antitumor response independent of antigens and that tumour-specific T-cell populations can be expanded during infection [11,12]. Still, studies also report increased cancer prevalence and increased cancer-related deaths in patients with infections [13,14]. Thus, this topic is still under debate and further research is necessary to better understand the underlying mechanisms and interaction of the simultaneous immune responses and their importance for the respective diseases.

In the present study, we demonstrate that influenza A virus (IAV) infection unexpectedly enhanced the anti-tumour immune response by increasing the activation of tumour-specific CD8^+^ T-cells, which reduced the tumour burden. The time of infection seems to be crucial for the activation of the anti-tumour immune response. Our results help to understand immune mechanisms in the context of concomitant challenges.

## Results

### Influenza A virus infection facilitates tumour control

**Fig 1:**
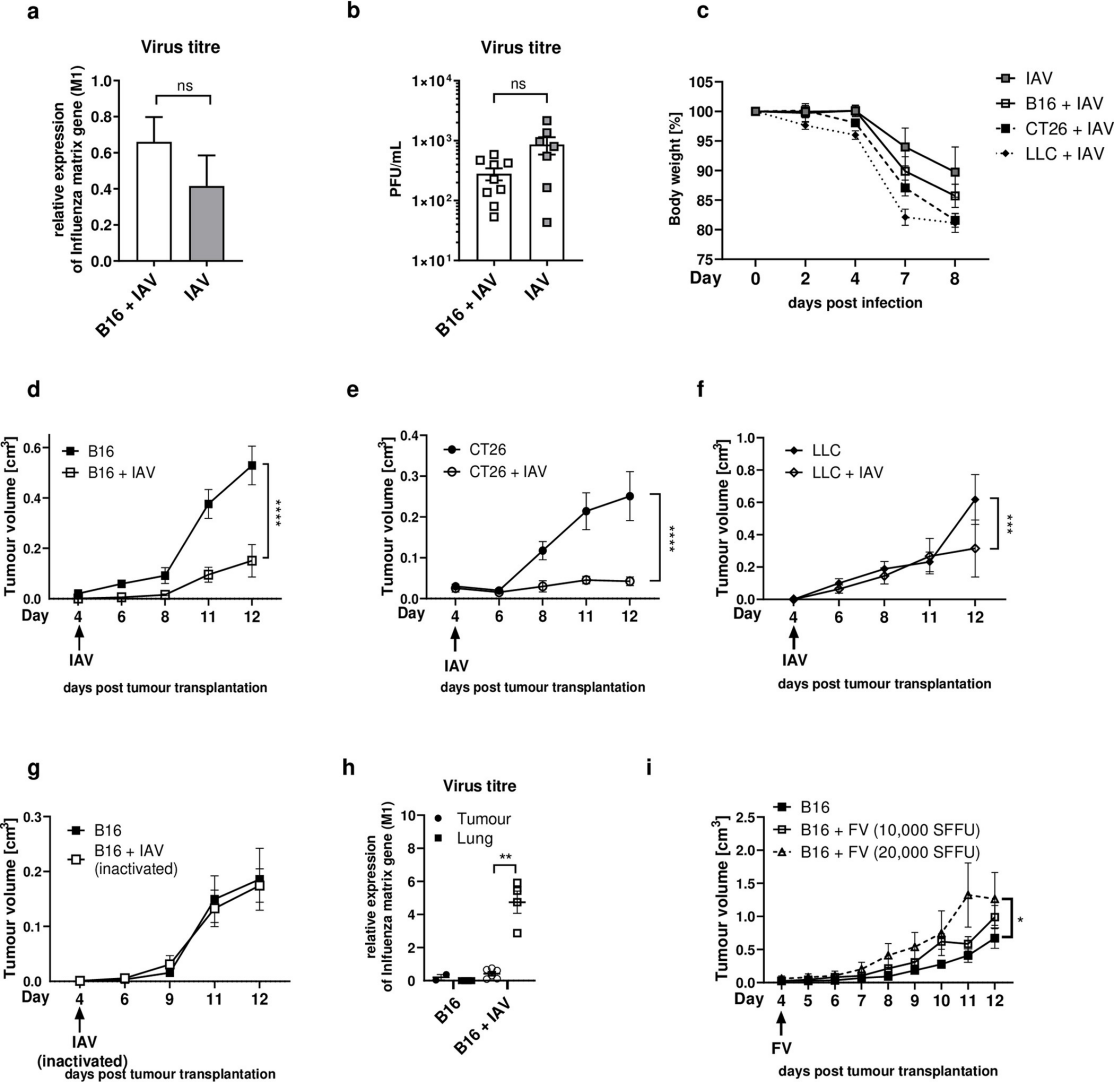
IAV infection suppresses tumour growth. **(a-d)** 1 x 10^5^ B16F1 (B16) tumour cells were transplanted subcutaneously (s.c.) into the right flank per mouse. 4 days after tumour transplantation, mice were infected intranasally with 150 PFU/mL Influenza A/PR/8/34 (IAV). (**a**) Viral load was measured by M1 expression relative to RPS9 expression in the lungs of infected mice with or without B16 tumour 8 days post infection (dpi). n = 9 for B16+IAV, n = 6 for IAV (**b**) Viral load as measured from homogenized lung supernatants by counting plaque forming units (PFU) from plaque assay 8 dpi. (**c**) Body weight change after IAV infection. Data from 2 experiments with 4–6 mice per group per experiment. **(d-g)** 1 x 10^5^ B16 (**d** and **g**), CT26 (**e**) or 5 x 10^5^ Lewis Lung Carcinoma (LLC) (**f**) tumour cells were transplanted s.c. and mice were infected as described in **a)** with (**d-f**) IAV or (**g**) inactivated IAV. The tumour volume was measured everyday once palpable upon infection. d&e: Data from 4 experiments with 3–4 mice per group per experiment, f: n = 4, g: Data from 2 experiments with 4 mice per group per experiment. (**h**) Viral load as measured by M1 expression relative to RPS9 expression in the tumour or lung of infected mice 12 days post tumour transplantation. (**i**) B16 tumours were transplanted s.c. with 1 x 10^5^ tumour cells and mice were infected intravenously with Friend Virus (FV) 4 days post tumour transplantation. The tumour volume was scored everyday once palpable. n = 4. Error bars represent SEM. Statistical tests on tumour growth development were performed as Two-way-ANOVA, in Sidak’s multiple comparisons test when two groups were compared or Tukey’s multiple comparisons test when more than two groups were compared. Viral titres were compared with non-parametric t tests. * = p<0.05, ** = p<0.01, *** = p<0.001, **** = p<0.0001, ns = not significant.

First, we determined the effect of tumour growth on IAV control. B16F1 (B16) melanoma cells were transplanted subcutaneously (s.c.) into the right flank of wildtype (WT) C57BL/6 mice and four days later, mice were infected intranasally with IAV. The onset of tumour growth did not influence IAV replication, as lung viral titres were unaffected by the presence or absence of a tumour 8 days after infection (Fig 1A and 1B). Furthermore, tumours did not induce a more severe disease development as indicated by no differences in weight loss progression compared to non tumour-bearing mice (Fig 1C). On the other hand, interestingly we did observe an effect of IAV infection on tumour growth. Strikingly, concomitant IAV infection significantly decreased tumour burden as tumours of infected mice were smaller at 8 days post infection (dpi) (Ø 0.151 cm^3^) compared to tumours of non-infected mice (Ø 0.483 cm^3^) (Figs 1D and S1a). Of note, tumour growth was not further restricted by low or high dose infection (S2 Fig). This antitumoural effect was not only limited to B16 tumours as also s.c. transplanted CT26 colon or Lewis Lung Carcinoma (LLC) tumours had significantly smaller sizes 8 days after IAV infection (Figs 1E, 1F, S1b, and S1c). To define whether acute infection with ongoing viral replication is required for the delay in tumour growth, mice were treated intranasally with inactivated IAV. However, treatment with inactivated IAV did not alter the tumour burden (Figs 1G and S1d), demonstrating that viral replication and the events of active inflammation of the lung are required for the observed tumour-controlling effect. Importantly, IAV infection was not associated with oncolytic activity as we were not able to detect viral RNA or infectious virus in tumours of infected mice (Fig 1H). Interestingly, respiratory infection seems to be crucial for the observed anti-tumour effect, as systemic infection with Friend retrovirus (FV), infecting nucleated erythroid precursors, did not induce tumour growth reduction. On the contrary, tumour growth increased significantly by FV infection and the severity of infection also increased in tumour-bearing mice, indicated by FV infection-induced splenomegaly (Figs 1I and S3). Thus, these results demonstrate that locally confined viral infection of the lungs with IAV during cancer did control tumour growth.

### Tumour growth control is dependent on CD8^+^ T-cells

**Fig 2:**
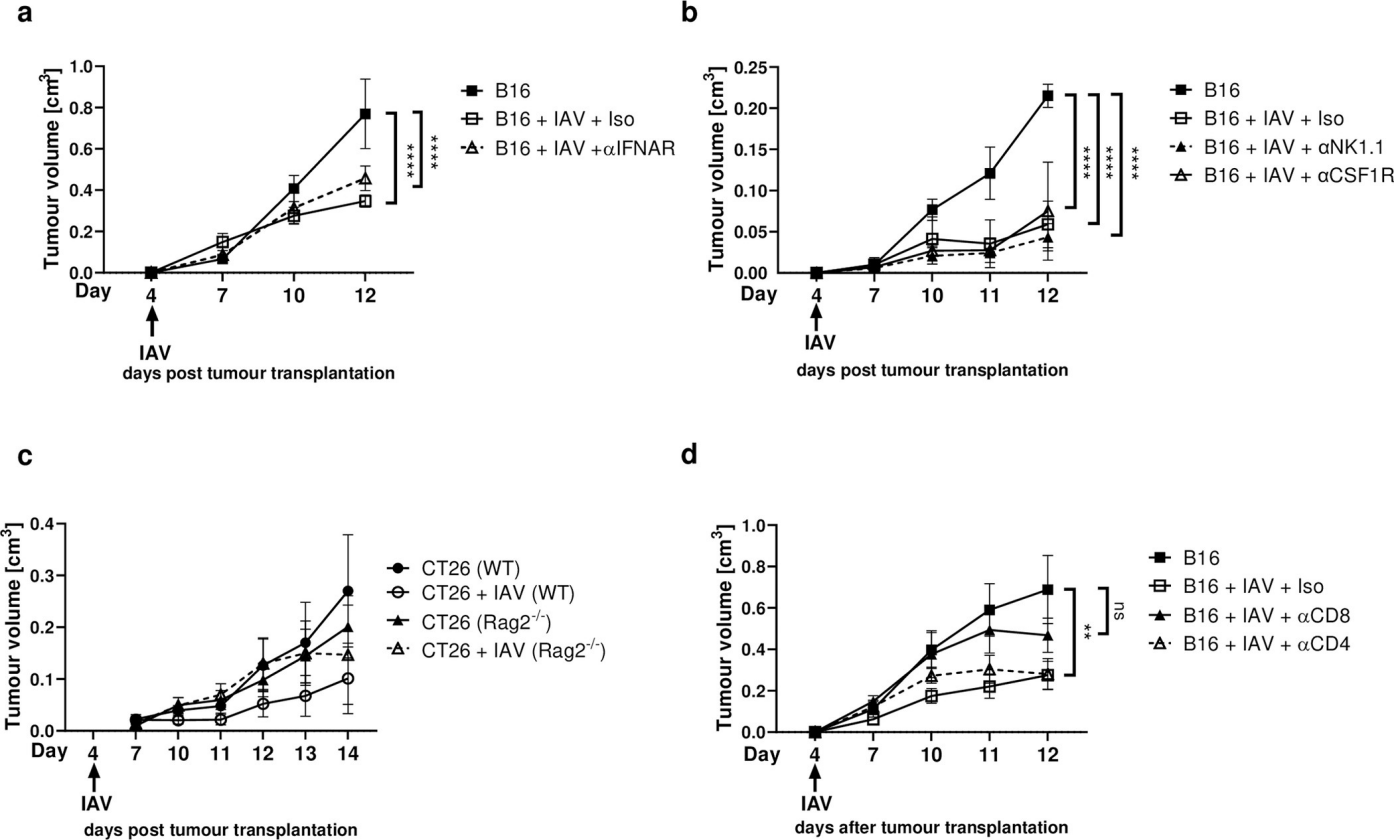
CD8^+^ T-cells are essential for tumour growth suppression during IAV infection. Tumours were subcutaneously (s.c.) transplanted and mice were infected with 150 PFU/mL Influenza A/PR/8/34 (IAV) as described. (**a**) 200 μg anti-IFNAR antibody was applied intraperitoneally (i.p.) on days 4, 7 and 10. n = 6. (**b**) For Natural Killer (NK)-cell or macrophage depletion 200 μg of antibodies against NK1.1 or CSF1R were applied i.p. respectively on days 4, 7 and 10. n = 3. (**c**) Rag2^-/-^ or BALB/c wildtype mice were s.c. injected with 1 x 10^5^ CT26 tumour cells followed by IAV (150 PFU/mL) infection. Data from 2 experiments with 3–4 mice per group per experiment. (**d**) For selective T-cell depletion 200 μg of antibodies against CD4 or CD8 were applied i.p. on days 4, 7 and 10. Data from 2 experiments with 4 mice per group per experiment. Error bars represent SEM. Statistical test on tumour growth development was performed as Two-way-ANOVA followed by Tukey’s multiple comparisons test. ** = p<0.01, **** = p<0.0001.

Since treatment with inactivated IAV did not alter tumour growth, active inflammatory processes appear to be involved in the restriction. An IAV infection leads to strong cytokine release, often referred to as “cytokine storm”. One prominent cytokine group among these are interferons (IFNs). IFN-α and IFN-β belong to the type I IFNs, bind both to the same interferon-alpha-receptor (IFNAR) and have been demonstrated to strongly influence the immune response and to inhibit tumour growth directly [15,16]. To determine the impact of type I IFNs on tumour control induced by IAV infection, tumour-bearing mice were treated with IFNAR-blocking antibodies during IAV infection. In fact, IFNAR blockade did not prevent control of tumour growth in IAV-infected mice (Fig 2A). This suggests that the cellular immune response may be more responsible for this effect. To determine which cell type is crucial for the reduced tumour growth upon IAV infection, we depleted specific immune cells. However, tumour growth control appears to be independent of Natural Killer (NK) cells and macrophages (MФ), as tumour growth was still severely restricted by IAV infection despite their respective depletion (Figs 2B, S4a, and S4b). To check whether the effect is mediated by cells of the adaptive immune systems, we repeated these experiments in Rag2^-/-^ mice, which are deficient in mature B- and T-cells. Indeed, adaptive immunity seems to be essential for the IAV induced effects as tumour-bearing Rag2^-/-^ mice did not show tumour growth control after IAV infection in contrast to infected tumour-bearing wildtype mice (Fig 2C). Therefore, we subsequently depleted CD8^+^ or CD4^+^ T-cells in tumour-bearing mice during IAV infection to test which of these cell types are the driver of tumour growth control in this setting. Importantly, depletion of CD8^+^ T-cells, but not CD4^+^ T-cells abrogated the effect of decreased tumour growth after IAV infection (Figs 2D, S4c, and S4d). Therefore, our data clearly indicate that CD8^+^ T-cells are required for the restriction of tumour growth.

### Cytotoxic CD8^+^ T-cells in the tumour are stronger activated upon lung infection

**Fig 3:**
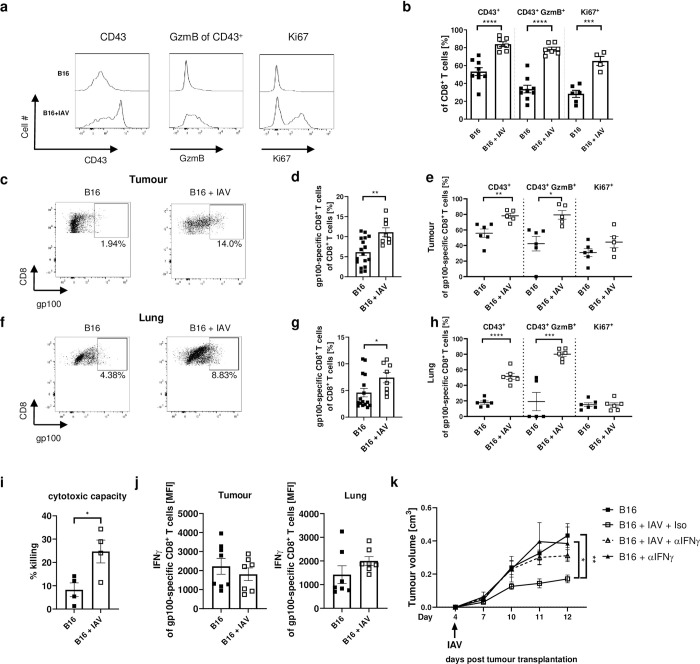
IAV infection strengthens cancer-specific CD8^+^ T-cell immunity. B16 tumour-bearing mice were infected with IAV (150 PFU/mL) as described. 8 days post infection mice were sacrificed for flow cytometry analysis. (**a**) Representative histograms of analysis of CD43, Granzyme B (GzmB), and Ki67 expression in the tumour. (**b**) Frequencies of CD43, GzmB of CD43^+^ or Ki67 expressing CD8^+^ T-cells in the tumour. (**c**) Representative dot plots of gp100-tetramer^+^ CD8^+^ T-cells in the tumour. (**d**) Frequencies of gp100-specific CD8^+^ T-cells in the tumour. (**e**) Frequencies of CD43, GzmB of CD43^+^ or Ki67 expressing gp100-specific CD8^+^ T-cells in the tumour. (**f**) Representative dot plots of gp100-tetramer^+^ CD8^+^ T-cells in the lung. (**g**) Frequencies of gp100-specific CD8^+^ T-cells in the lung. (**h**) Frequencies of CD43, GzmB of CD43^+^ or Ki67 expressing gp100-specific CD8^+^ T-cells in the lung. (**i**) CD8^+^ T cells were isolated from tumours 12 days after tumour transplantation and co-cultured with eFluor670 stained B16 cells. After 18 h of co-culture, the frequency of remaining viable tumour cells was quantified using flow cytometry, and cytotoxic capacity was calculated in comparison to tumour cell-only wells (n  =  4 co-cultures per group). (**j**) Mean fluorescence intensity (MFI) of Interferon (IFN)γ expressing gp100-specific CD8^+^ T-cells. (**k**) B16 tumour-bearing mice were treated with 250 μg anti-IFNγ antibodies on days 4, 6, 8 and 10 post tumour cell injection. Data from 2 experiments with 4 mice per group per experiment. Error bars represent SEM. Statistical tests between two groups were performed as Student’s t-tests. Statistical test on tumour growth development was performed as Two-way-ANOVA, followed by Tukey’s multiple comparisons test. * = p<0.05, ** = p<0.01, *** = p<0.001, **** = p<0.0001.

After identifying CD8^+^ T-cells as key regulators for the tumour growth, we characterized the phenotype of the CD8^+^ T-cells in the tumour in more detail. Interestingly, upon infection CD8^+^ T-cells in the tumour showed enhanced activation and proliferation, indicated by the stronger expression of CD43 and Ki67 compared to non-infected tumour-bearing mice (Fig 3A and 3B). We also observed a significantly higher frequency of strongly activated CD43^+^ CD8^+^ T-cells expressing the cytotoxic molecule granzyme B (GzmB) in tumours of IAV-infected mice (Fig 3A and 3B). To check for tumour-specificity of CD8^+^ T-cells, we used a labelled MHC-tetramer directly binding to the respective T-cell receptor against the melanoma antigen gp100. Of note, the frequency of gp100-specific CD8^+^ T-cells was significantly increased in the tumour after IAV infection compared to non-infected tumour-bearing mice (Fig 3C and 3D). In this regard, we found significantly increased proportions of highly activated CD43^+^ gp100-specific CD8^+^ T-cells that also expressed GzmB when the tumour-bearing animals were infected with IAV (Fig 3E). Frequencies of gp100-specific CD8^+^ T-cells were also significantly increased in the lung of IAV-infected tumour-bearing mice expressing significantly higher levels of CD43 and GzmB compared to CD8^+^ T-cells from non-infected mice (Fig 3F–3H). To test whether CD8^+^ T-cells from the tumour of infected mice also exhibit increased antitumor cytotoxicity, we isolated CD8^+^ T-cells from the tumour of infected and uninfected mice and incubated them *ex vivo* with fluorescently labelled B16 cells. In fact, CD8^+^ T-cells from the tumour of infected mice had a higher cytotoxic capacity compared to CD8^+^ T-cells from uninfected mice, as fewer viable B16 cells were detectable after 18 h (Fig 3I). This demonstrates that the tumour-specific CD8^+^ T-cells actually act more cytotoxic due to the IAV infection. The tumour-specific CD8^+^ T-cells also showed increased expression of IFNγ in the lung of IAV-infected tumour-bearing mice. Although expression of IFNγ by tumour-specific CD8^+^ T-cells in the tumour was not altered (Fig 3J), direct IFNγ neutralization during IAV infection of tumour-bearing mice abolished the positive effect (Fig 3K).

**Fig 4:**
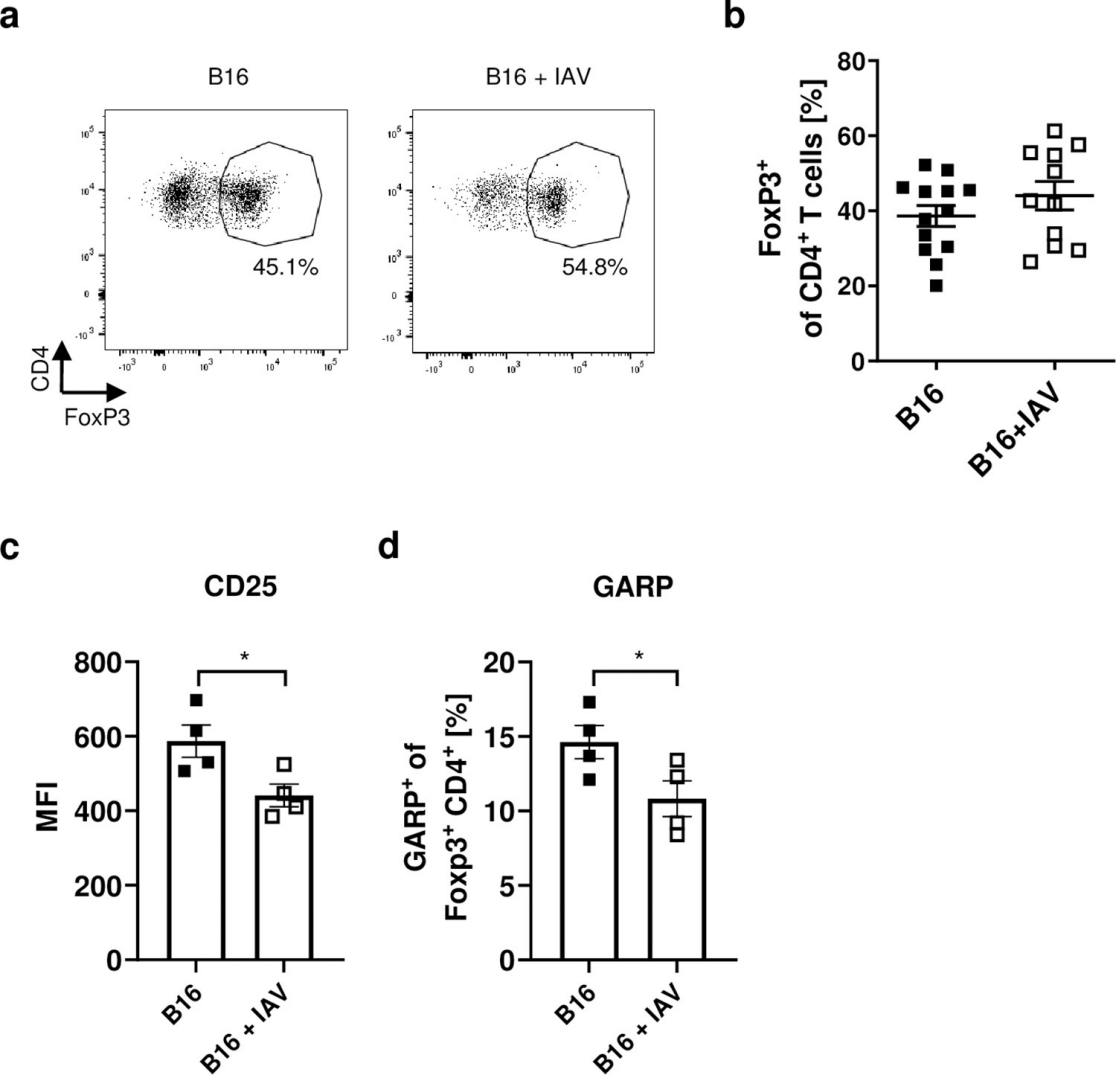
IAV infection reduces activation of Tregs. B16 tumour-bearing mice were infected with IAV as described. 8 days post infection mice were sacrificed for flow cytometry analysis. (**a**) Representative dot plots of CD4^+^ T-cells expressing FoxP3. (**b**) Frequencies of FoxP3^+^ of CD4^+^ T-cells in the tumour. Data from 3 experiments are shown. (**c**) Mean fluorescent intensity (MFI) of CD25 of FoxP3^+^ CD4^+^ T-cells in the tumour. (**d**) Frequencies of GARP expressing FoxP3^+^ CD4^+^ T-cells. Error bars represent SEM. Statistical tests between two groups were performed as Student’s t-tests. * = p<0.05.

In contrast to CD8^+^ T-cells, we could not detect any differences in the proportion and activation of CD4^+^ T-cells in the tumour between infected and uninfected mice (S5a and S5b Fig). In addition, frequencies of CD4^+^ regulatory T-cells (Tregs) in the tumours were unaltered (Fig 4A and 4B). However, we detected decreased expression of CD25 and the transmembrane protein glycoprotein A repetitions predominant (GARP) on Tregs in the tumour from infected mice compared to uninfected mice (Fig 4C and 4D). CD25 acts as an IL-2 receptor, so that lower CD25 expression reduces the competition for IL-2 with CD8^+^ T-cells [17]. GARP, in turn, is important for the release of immunosuppressive TGF-β [18]. Taken together, these data suggest lower activation of Tregs, which is in good agreement with increased CD8^+^ T-cell activation.

Likewise, we were also able to measure IAV-specific CD8^+^ T-cells in the tumour 8 dpi. Noticeably, the activation phenotype of these nucleoprotein (NP)-specific CD8^+^ T-cells in the tumour was comparable to those in the lungs in infection (S6a Fig). However, the influence of tumour-infiltrating IAV-specific CD8^+^ T-cells on tumour growth was negligible, as transferred CD8^+^ T-cells from infected lungs into non-infected tumour-bearing mice did not restrict tumour growth, neither via i.v. adoptive cell transfer (S6b Fig), nor by direct injection into the tumour (S6c Fig). This suggests that the tumour growth restriction caused by IAV infection is rather not due to a bystander T-cell effect, but is rather tumour antigen-specific.

### IAV infection expands tumour-specific PD-1^int^ TIM-3^-^ CD8^+^ T-cells in the tumour

**Fig 5:**
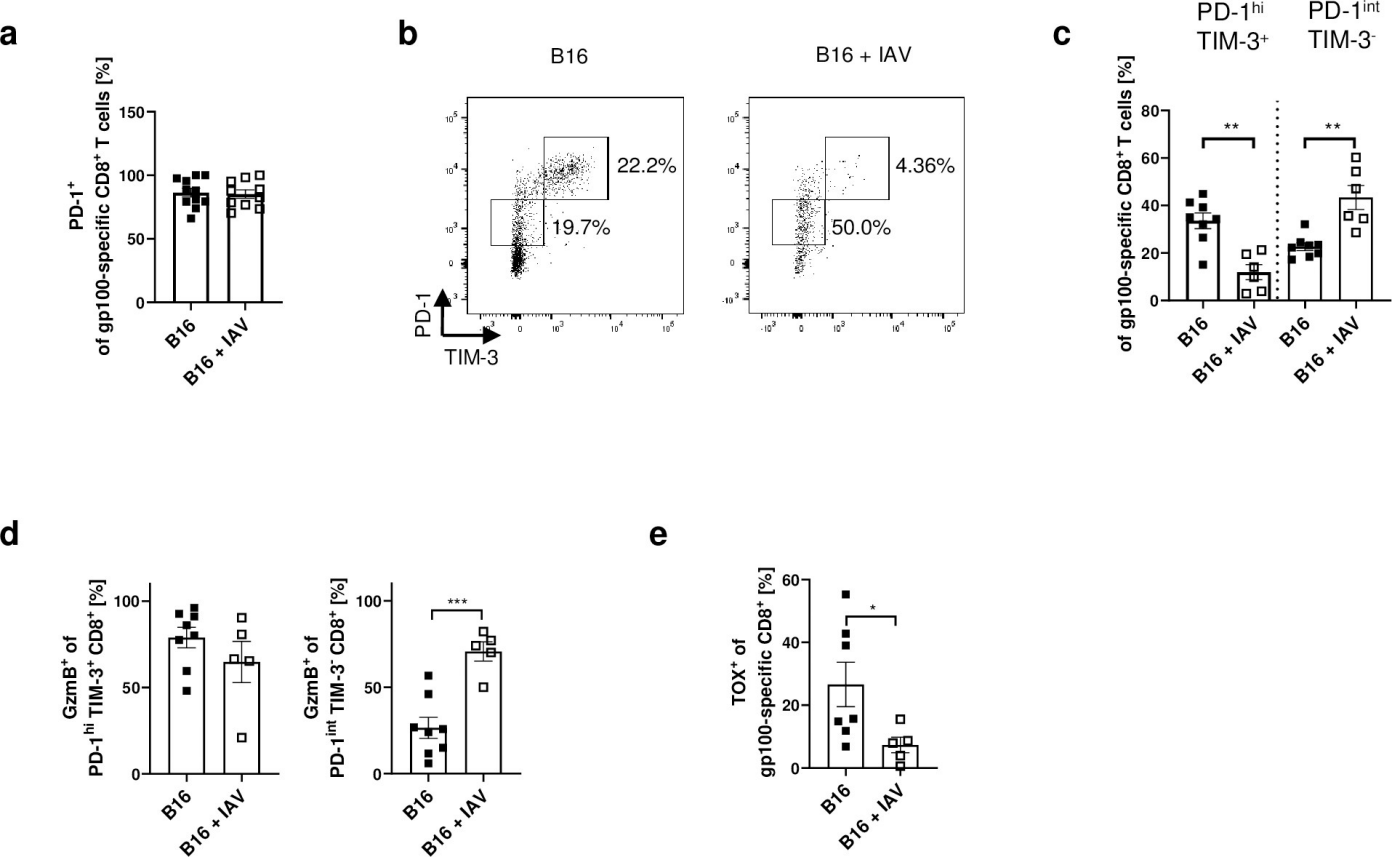
IAV infection impedes differentiation of T-cell exhaustion. B16F1 (B16) tumour-bearing mice were infected with Influenza A/PR/8/34 (IAV) (150 PFU/mL) as described. 8 days post infection mice were sacrificed for flow cytometry analysis. (**a**) Frequencies of PD-1 expressing gp100-specific CD8^+^ T-cells in the tumour. (**b**) Representative dot plots of CD8^+^ T-cells expressing PD-1 and TIM-3. (**c**) Frequencies of PD-1^high^ TIM-3^+^ or PD-1^int^ TIM-3^-^ of gp100-specific CD8^+^ T-cells in the tumour. (**d**) Frequencies of Granzyme B (GzmB) expressing cells of the populations described in c. (**e**) Frequencies of Thymocyte selection-associated high mobility group box protein (TOX) expressing gp100-specific CD8^+^ T-cells in the tumour. Data from 2 experiments are shown. Error bars represent SEM. Statistical tests between two groups were performed as Student’s t-tests. * = p<0.05, ** = p<0.01, *** = p<0.001.

The tumour-specific CD8^+^ T-cells in the TME showed no difference in expression of PD-1 (Fig 5A). However, we detected differences in PD-1^+^ populations co-expressing TIM-3 after IAV infection (Fig 5B and 5C). PD-1^hi^ TIM-3^+^ T-cells have been described to be highly dysfunctional compared to PD-1^int^ TIM-3^-^ CD8^+^ T-cells in tumours [19]. Interestingly, while the PD-1^hi^ TIM-3^+^ population in the tumour showed no difference in GzmB expression with and without infection, we found a significantly increased proportion of GzmB-expressing PD-1^int^ TIM-3^-^ CD8^+^ T-cells (Fig 5D). In general, T-cell exhaustion appeared to be more advanced in tumour-specific CD8^+^ T-cells from non-infected mice, as indicated by a higher proportion of the Thymocyte selection-associated high mobility group box protein (TOX)-expressing tumour-specific CD8^+^ T-cells compared to infected mice (Fig 5E). Thus, the data suggest that T-cell exhaustion differentiation is impaired by IAV infection.

### IAV infection induces the migration of tumour-specific CD8^+^ T-cells to the lung

**Fig 6:**
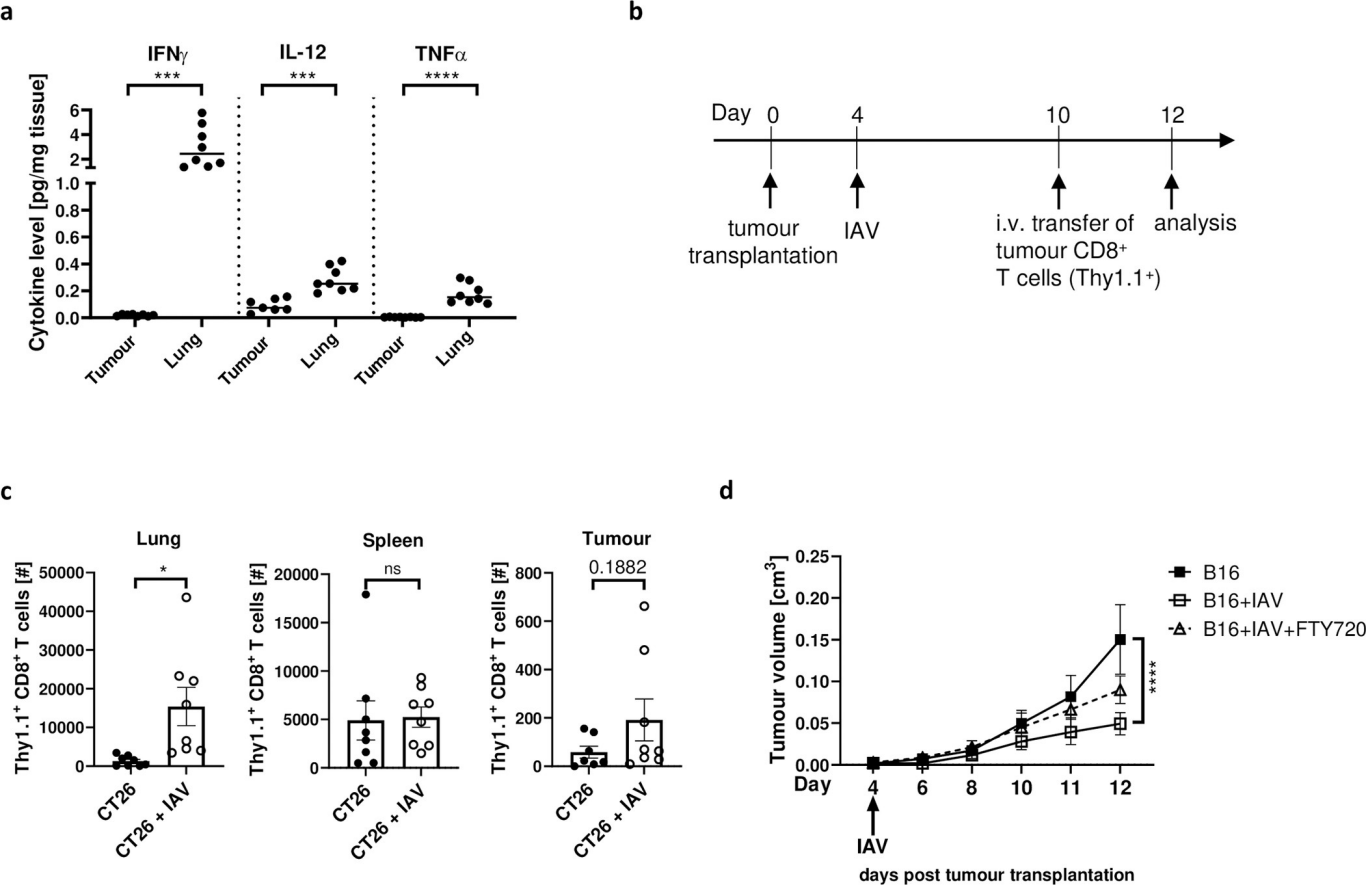
Tumour-specific CD8^+^ T-cells are recruited to the IAV-infected lung. (**a**) Cytokine concentrations in the tumour and lung were analysed by Luminex analysis 12 days post tumour cell injection in mice infected with IAV. (**b**) CT26 tumours were transplanted into BALB/c mice, which were then infected with Influenza A/PR/8/34 (IAV). CT26 tumour derived CD8^+^ T-cells from Thy1.1 mice were adoptively transferred into infected and non-infected mice. (**c**) Two days after adoptive transfer, the numbers of Thy1.1^+^ CD8^+^ T-cells were analysed in the lung, spleen and tumour. (**d**) B16 tumour-bearing mice were infected with IAV (150 PFU/mL) as described. Mice were treated with FTY720 every second day upon infection to avoid lymphocyte egress from lymphoid organs. Data from 2 experiments with 4 mice per group per experiment. Error bars represent SEM. Statistical tests between two groups were performed as Student’s t-tests. Statistical tests on tumour growth development were performed as Two-way-ANOVA, in Tukey’s multiple comparisons test. * = p<0.05, *** = p<0.001, **** = p<0.0001. ns = not significant.

Since we could identify an increased infiltration of the lung by tumour-specific CD8^+^ T-cells after IAV infection, we investigated to what extent the infection in the lung could modulate the phenotype of tumour-specific CD8^+^ T-cells. We detected a stronger pro-inflammatory environment in the lungs than in tumours of IAV-infected tumour-bearing mice, indicated by increased IFNγ, IL-12 and TNFα concentrations (Fig 6A). Therefore, we hypothesized that tumour-specific CD8^+^ T-cells might be recruited into the lungs during infection, getting bystander activated in the inflamed lung, and then migrate back into the tumour where they control tumour growth. To investigate the migration of tumour-derived CD8^+^ T-cells *in vivo*, Thy1.1^+^ CD8^+^ T-cells were isolated from tumours of congenic Thy1.1 mice and adoptively transferred into infected (6 dpi) or non-infected tumour-bearing wildtype mice (Fig 6B). While no differences were observed in the number of transferred Thy1.1^+^ CD8^+^ T-cells in the spleen between infected and non-infected mice, both the tumour and more prominently the lung showed an increased infiltration by transferred Thy1.1^+^ CD8^+^ T-cells upon additional infection compared to non-infected mice (Fig 6C). To confirm that the migration of CD8^+^ T-cells into the tumour is indeed important for the IAV-mediated antitumor response, we treated non-infected and infected tumour-bearing mice with FTY720, which is an inhibitor of lymphocyte emigration from lymphoid organs. In line with the observed dependence on CD8^+^ T-cells, tumour growth delay was abrogated when lymphocyte egress was inhibited by the application of FTY720, leading to bigger tumours in infected mice compared to untreated infected tumour-bearing mice (Fig 6D). These observations indicate the migration and need for *de novo* infiltration of CD8^+^ T-cells into the TME during IAV infection to restrict tumour growth, rather than boosting pre-existing CD8^+^ T-cell response directly in the TME.

### CXCR3 is important for the recruitment of tumour-specific CD8^+^ T-cells to the infected lung and maintenance of the enhanced antitumor response

**Fig 7:**
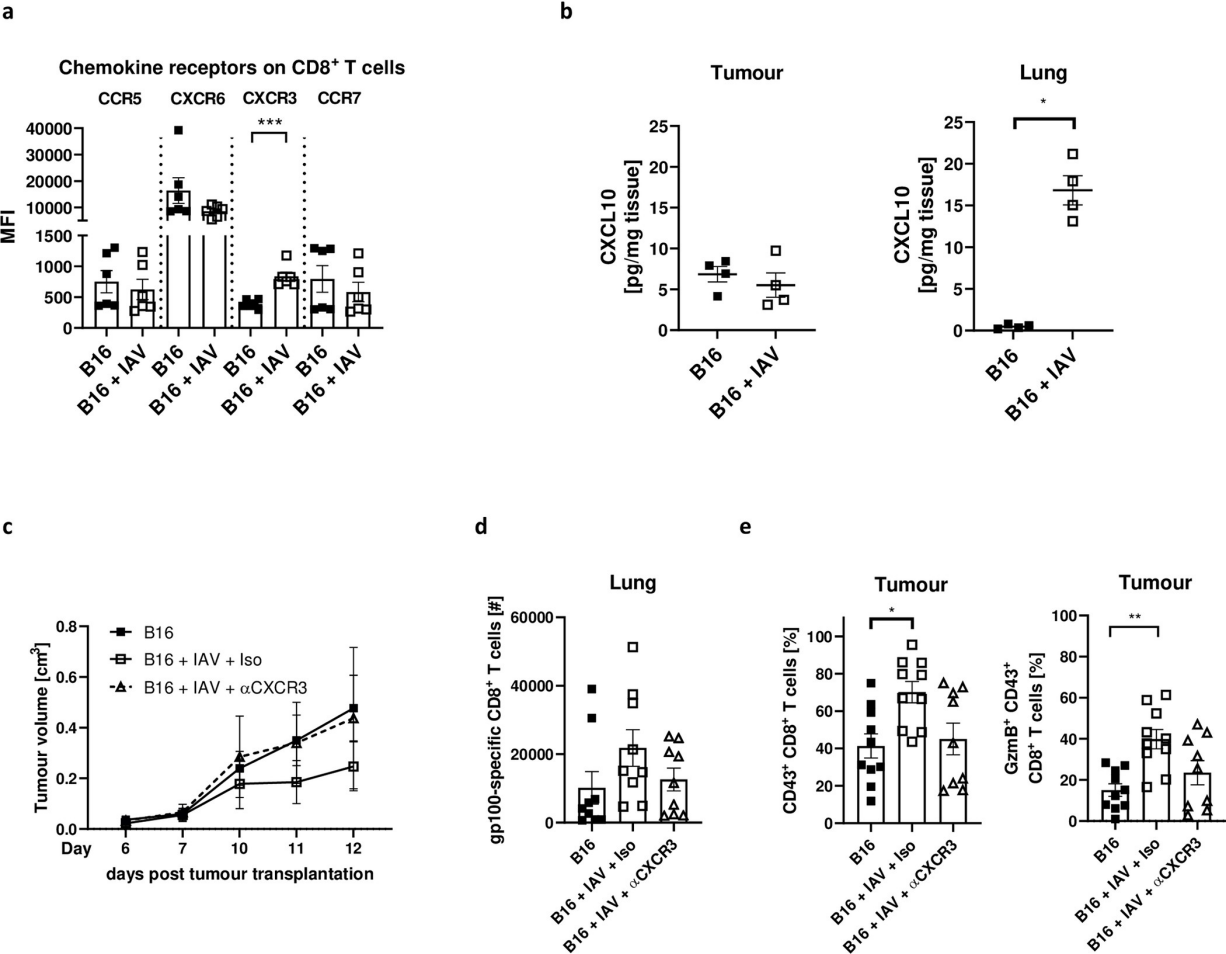
Blockade of CXCR3 reverses tumour growth restriction by IAV infection. B16 tumour-bearing mice were infected with Influenza A/PR/8/34 (IAV) (150 PFU/mL) as described. 8 days post infection mice were sacrificed for flow cytometry or Luminex analysis. (**a**) Chemokines were analysed via flow cytometry. Mean fluorescence intensity (MFI) of the respective chemokine receptors was measured on CD8^+^ T cells in the tumour. Data from two individual experiments are shown. (**b**) Absolute CXCL10 levels in tumour and lung were measured by Luminex analysis 12 days post tumour cell injection. (**c**) B16 tumour-bearing mice were infected with IAV and treated with 200 μg anti-CXCR3 antibodies on days 4, 7 and 10 post tumour cell injection. Data from 3 experiments with 3–4 mice per group per experiment. (**d**) Absolute cell numbers of gp100-specific CD8^+^ T-cells in the lung from mice treated as described in **c)**. (**e**) Frequencies of CD43 and Granzyme B (GzmB) expressing CD8^+^ T-cells in the tumour. Error bars represent SEM. Statistical tests between two groups were performed as non-parametric Mann-Whitney t-tests. Statistical tests with more than two groups were performed as One-way-ANOVA, followed by Kruskal-Wallis multiple comparison test. Statistical tests on tumour growth development were performed as Two-way-ANOVA, in Tukey’s multiple comparisons test. * = p<0.05, ** = p<0.01.

Since chemokines are important players for the migration of immune cells through the body, we screened CD8^+^ T-cells for the expression of various chemokine receptors. Among different chemokine receptors examined, we found CXCR3 significantly increased expressed on CD8^+^ T-cells in the tumour (Fig 7A). In line with this, the CXCR3 ligand CXCL10 was significantly increased in the lungs of IAV-infected animals (Fig 7B). Likewise, CXCL9 and CXCL11, the two other ligands of CXCR3, were enhanced in infected lungs (S7 Fig). Hence, CD8^+^ T-cells could be recruited into the lungs using the CXCR3:ligand axis. To test this, CXCR3 was blocked in infected tumour-bearing mice using a monoclonal antibody. Importantly, this resulted in reversal of tumour growth restriction compared to infected animals without CXCR3 blockade (Fig 7C). At the same time, we found fewer tumour-specific CD8^+^ T-cells in the lungs, and lower expression of the activation marker CD43 and GzmB in CD8^+^ T-cells in the tumour when CXCR3 was blocked compared to untreated IAV infected tumour bearing mice (Fig 7D and 7E). Thus, our data demonstrate that CXCR3 is important for recruiting CD8^+^ T-cells from the tumour in order to get activated in the lungs.

### Timing of IAV infection is critical for reduced tumour growth

**Fig 8:**
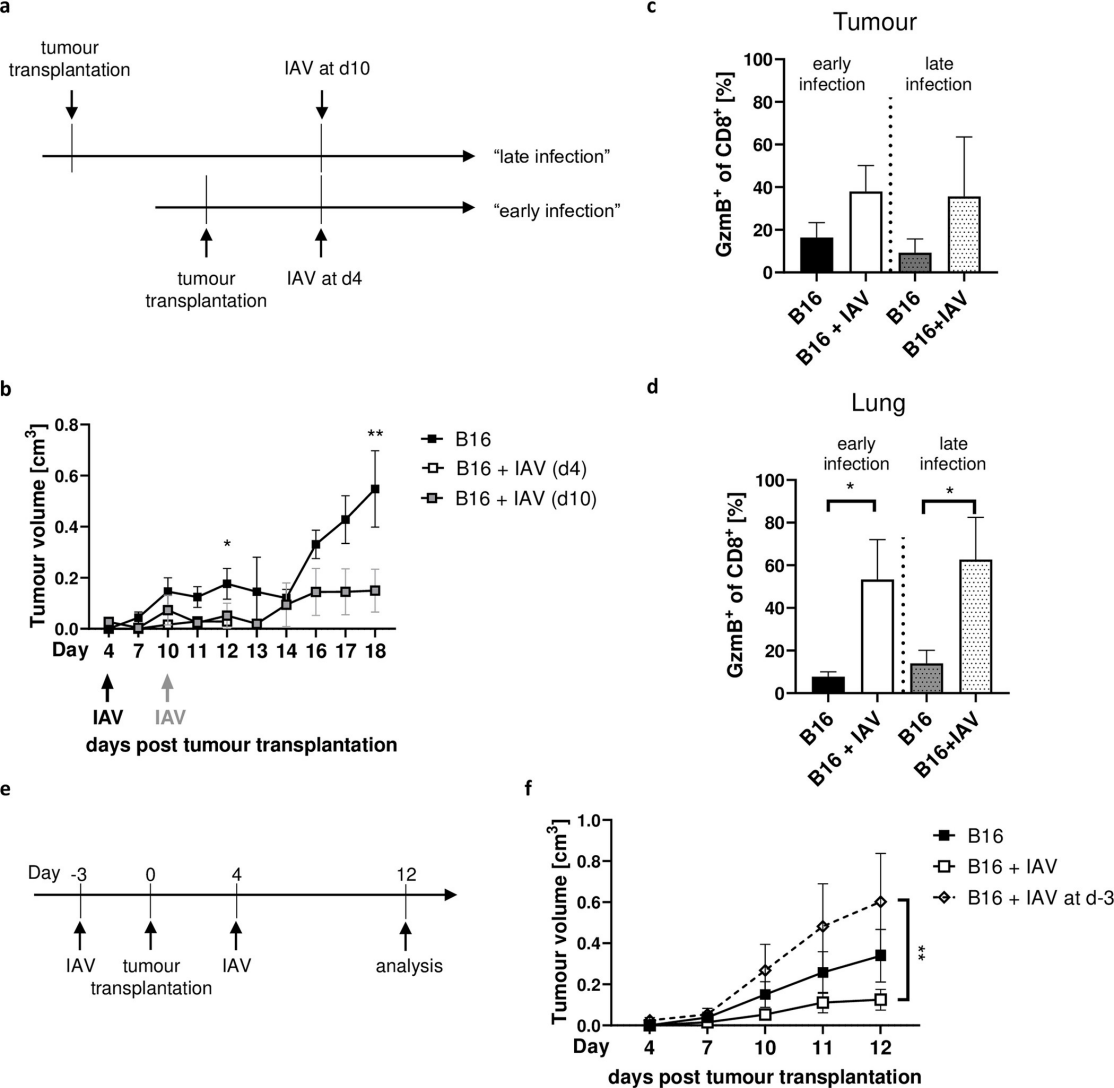
Infection at a later timepoint still enables tumour growth restriction but previous infection reverses the effect. (**a**) B16F1 (B16) tumour cells were injected into mice. Influenza A/PR/8/34 (IAV) infection followed either 4 days (“early infection”) or 10 days (“late infection”) post tumour transplantation. (**b**) Tumour growth is shown. Data from 2 experiments with 3–4 mice per group per experiment. (**c**) Frequencies of Granzyme B (GzmB) expressing CD8^+^ T-cells in the tumour 18 (late) or 12 (early) days post tumour cell injection. (**d**) Frequencies of GzmB expressing CD8^+^ T-cells in the lung 18 (late infection) or 12 (early infection) days post tumour cell injection. (**e**) Mice were infected 3 days before (d-3) or 4 days post tumour transplantation. Data from 2 experiments with 4 mice per group per experiment. Error bars represent SEM. Statistical tests between two groups were performed as Student’s t-tests. Statistical tests on tumour growth development were performed as Two-way-ANOVA, followed by Tukey’s multiple comparisons test. * = p<0.05, ** = p<0.01.

To investigate whether IAV infection decreases tumour growth also at later stages of tumour development, we infected mice at day 10 post tumour cell transplantation (Fig 8A). Importantly, tumour growth was significantly decreased both when mice were infected 4 or 10 days post tumour cell transplantation compared to non-infected tumour-bearing mice (Fig 8B). GzmB expression by tumour-specific CD8^+^ T-cells in tumours and lungs were similarly enhanced when mice were infected at day 4 or 10 post tumour cell transplantation (Fig 8C and 8D). However, tumour cell transplantation into acutely IAV-infected mice showed opposite effects, i.e. even increased tumour growth (Fig 8E and 8F). Thus, IAV infection after tumour onset is mandatory for its enhancing antitumor effect.

## Discussion

In this work, we delineate a relationship between respiratory viral infection and anti-tumour effects in locally distinct tumours. Using tumour transplantation mouse models for different tumour types, we propose that in case of respiratory infection, tumour-specific CD8^+^ T-cells are able to migrate outside the TME in a CXCR3 dependent manner to become highly activated in the lungs, so that they ultimately help to reduce tumour growth. Elucidating the detailed mechanisms by which respiratory viral infection remodulates the tumour-specific immune response will greatly advance our fundamental understanding of how the immune system responds to concomitant challenges.

We demonstrate that IAV infection during cancer disease enabled the host’s immune response to significantly slow down the growth of various types of solid tumours. A previous study described that IAV infection-induced immune responses can lead to the induction of T-cells specific for tumour-associated self-antigens (TAA) and thus mediate reduced tumour growth in case of cancer [12]. This indicates an important cross talk of anti-viral and anti-tumour immune response. Studies in healthy individuals revealed a repertoire of already existing TAAs, including TAAs associated with melanoma [20]. These findings are well in line with our data since we observed that IAV infection led to increased activation of CD8^+^ T-cells in both tumour and lung. The treatment with inactivated virus does not show the same effect indicating that effective T-cell priming is necessary for T-cell activation. Local viral infection of the lung seems to be important for this immune modulation as a systemic FV infection did not inhibit tumour growth. However, it should be taken into account that FV infection has been shown to induce immunosuppression [21], e.g. IL-10 or Treg levels increase by FV and by SFFV in particular [22]. This could partially explain why FV infection does not result in the same anti-tumour response as IAV infection. This therefore underlines that the observed effect is IAV-specific, as we did not detect any Treg induction, for example. Furthermore, although FV infected DCs lose their APC function, B-cells increase their CTL stimulation [23] so that a CTL response against the tumour could still be possible. Indeed, B-cells can contribute to an anti-tumour response also in B16 melanoma [24]. The contribution of B-cells in our model remains to be further investigated. Although B16 melanoma cells seem to be *per se* permissive for IAV infection *in vitro* [25,26], IAV does not replicate in the tumour in our model. Therefore, the slowed tumour growth cannot be the result of direct tumour cell lysis by the virus, but is rather immune-mediated. This is also supported by the fact that the reduced tumour growth was exclusively reversed with the depletion of CD8^+^ T-cells and supports the thesis that enhanced activation of the anti-tumour CD8^+^ T-cell response is the main reason for tumour control. The contribution of cytotoxic CD8^+^ T-cells in tumour control is well established [27,28]. To characterise CD8^+^ T-cells in this study we focused on well established markers for activation (CD43^+^), cytotoxicity (GzmB^+^) and proliferation (Ki67^+^). CD8^+^ T-cells expressing the activation-associated glycosylated isoform CD43 are defined as potent effector T-cells [29]. We were able to demonstrate increased cytotoxicity of CD8^+^ T-cells from IAV-infected tumour-bearing mice using an *ex vivo* cytotoxicity test, an important prerequisite for tumour cell killing. Indeed, an elevated number of CD8^+^ T-cells in the TME seems to correlate with improved clinical outcome [30]. In addition to their cytotoxic activity, it has also been shown previously that CD8^+^ T-cells suppress angiogenesis with the help of IFNγ [31]. An increased number of activated cytotoxic CD8^+^ T-cells could also lead to an increased effect of CD8^+^ T-cell produced IFNγ and finally important for the reduction of tumour growth in infected animals, as we find that neutralization of IFNγ was able to reverse this effect. Even if other immune cells such as NK-cells or CD4^+^ cells can also produce IFNγ, a depletion of these cell types, in contrast to CD8^+^ T-cell depletion, did not lead to a reversal of the tumour growth reduction.

However, the TME is often of immunosuppressive nature by secretion of immunosuppressive cytokines [32] and enhancement of anti-immunogenic cells such as that of Tregs [33]. Especially melanoma is known for a strongly immunosuppressive microenvironment with very few activated CD8^+^ T-cells [34]. In our study, we detected a clear impact of lung infection on CD8^+^ T-cells in the TME. CD8^+^ T-cells were stronger activated with additional infection and in agreement with this, we observed a significant reduction in terminally exhausted TIM-3^+^ PD-1^high^ CD8^+^ T-cells in the tumour upon infection. This is also important as Kohlhapp *et al*. previously reported progressive exhaustion of tumour-specific CD8^+^ T-cells by influenza infection, indicated by increased PD-1 expression [35]. Although they described that IAV infection can accelerate tumour growth, we were surprisingly able to observe the opposite effect. Kohlhapp *et al*. used an experimental model of previous infection and subsequent tumour cell transplantation in contrast to our experimental setup. Although the aforementioned study does not provide any data on tumour growth, it does describe a reduced survival of infected compared to non-infected tumour-bearing mice. In line with this, we also observed an accelerating effect of the infection on tumour volume if mice were infected before tumour cell injection. Therefore, the effect of IAV infection on anti-tumour responses seems to be strongly dependent on the timing of infection highlighting the dynamic interaction of tumour- and infection-related immune responses. Clinically, an acute infection during the course of tumour growth is presumably the more common case compared to tumour manifestation during an acute infection. The infection dose also seems to have a distinct influence, since we have shown that infection with both lower and higher infectious doses does not result in increased tumour growth restriction. Our data therefore emphasizes the importance of a balanced overall immune response.

The strong activation of the tumour-specific CD8^+^ T-cell response observed by us after IAV infection could be due to ongoing inflammatory events in the lungs, since we were able to detect tumour-specific CD8^+^ T-cells there. This is supported by our observation that the transfer of tumour-derived CD8^+^ T-cells into infected tumour-bearing mice revealed an increased migration of these cells both into the lung and into the tumour in contrast to the transfer into non-infected tumour-bearing mice. We suggest that the migration from the anti-inflammatory TME milieu to the pro-inflammatory milieu in the inflamed lung leads to the observed stronger activation upon infection compared to non-infected mice. This is also supported by the observation that blocking the lymph node egress of T-cells by FTY720 application abrogates the reduced tumour growth in IAV-infected tumour-bearing mice. The chemokine receptor CXCR3 could possibly be of decisive importance for the migration of CD8^+^ T-cells. CXCR3 has been previously described as important for the recruitment of newly activated effector T-cells into inflamed tissue [36]. This can also be found in our data. Not only were we able to detect increased levels of CXCR3-expressing CD8^+^ T-cells after IAV infection in tumour-bearing mice, but also that blocking CXCR3 abolished the infection-induced effect of reduced tumour growth. Furthermore, we were able to detect elevated levels of CXCL10 in lung tissues after IAV infection. This may provide a gradient sufficient for tumour-specific CXCR3^+^ CD8^+^ T cells to migrate into the respiratory tract. In line with this result, both the number of tumour-specific CD8^+^ T-cells in the lung and CD8^+^ T-cell activation in the tumour were not increased after CXCR3 blockade. This indicates that CXCR3-mediated migration of CD8^+^ T-cells is of great importance. In this context, the reduced activation of Tregs could also lead to improved migration, as Tregs have been shown to coordinate the migration of effector T-cells by controlling inflammatory chemokine gradients [37]. In general Tregs are believed to significantly contribute to tumour immune evasion by hindering the efficacy of anti-tumour immune responses [38]. Thus, after IAV infection, impaired Tregs could contribute to the increased CD8^+^ T-cell-mediated anti-tumour effect.

Of note, the capacity of migration is not limited to the tumour-derived CD8^+^ T-cells but *vice versa* we also detected influenza-specific CD8^+^ T-cells in the tumour. However, their contribution to tumour growth control seems to be minor since, unlike indicated in the bacterial *L*. *monocytogenes* model [11], the transfer of IAV-specific CD8^+^ T-cells did not lead to diminished tumour growth. This suggests that the quality of bacterial and viral induced CD8^+^ T-cell responses is different.

In general, it is difficult to assess the effect of IAV infection on the immune response in cancer patients, since many of the patients are immunosuppressed by chemotherapy and tend to have a worse course of IAV infection [39]. Pre-existing antibody-based immunity against IAV might also prevent influenza infection [40]. This can potentially reduce the restrictive effect of the infection on tumour growth. Therefore, our study has a certain limitation, as our results cannot be fully replicated in humans in some respects. Nevertheless, it is fundamentally important to better understand the interaction between infection and cancer and to learn from this interaction in order to use this knowledge to improve cancer therapies.

In summary, our results provide new insights into the effect of respiratory infection on the anti-tumour CD8^+^ T-cell response. It is important to understand that the interaction of the two events, tumour disease and infection, is in constant motion and tumour-derived CD8^+^ T-cells do have the potential for strong activation if they are placed in an appropriate environment. This should be taken into account in new therapeutic approaches such as tumour-specific vaccination. Our data suggest that novel mucosal immunisation strategies that incorporate certain pro-inflammatory factors may have the potential to dramatically increase the success of effectively inducing tumour-specific T-cell responses, even against non-mucosal tumours.

## Methods

### Ethics statement

Animal experiments were performed in strict accordance with the German regulations of the Society for Laboratory Animal Science (GV-SOLAS) and the European Health Law of the Federation of Laboratory Animal Science Associations (FELASA). The protocol was approved by the North Rhine-Westphalia State Agency Landesamt für Natur, Uwelt und Verbraucherschutz (LANUV). All efforts were made to minimize suffering.

### Mice

Female wildtype C57BL/6J mice and BALB/c mice were purchased from Envigo Laboratories (Horst, Netherlands). Rag2^-/-^ BALB/c mice, and Thy1.1^+^ BALB/c and were bred in-house. Six- to eight-week old female mice were used for B16F1, CT26, or LLC studies. Mice were housed under specific-pathogen-free conditions in the Laboratory Animal Facility of the University Hospital Essen with 12-hour light/dark cycles, relative humidity of 55 ± 5%, and temperature of 21 ± 2°C.

### Cell culture

Cells were cultivated at 37°C in a humidified atmosphere of 5% CO_2_. B16F1 and CT26 tumour cells were grown in Iscove’s Modified Dulbecco’s Medium (IMDM) GlutaMAX, 25 mM Hepes (Invitrogen), supplemented with 10% heat-inactivated FCS (Sigma Aldrich), 2.5 μM β-Mercaptoethanol and 1% Penicillin/Streptomycin. LLC tumour cells were grown in Roswell Park Memorial Institute (RPMI) 1640 Medium with Glutamax, 25mM Hepes (Invitrogen), supplemented with 10% heat-inactivated FCS, 1% L-Glutamine and 1% Penicilline/Streptomycin. Madin-Darby canine kidney (MDCK) cells were grown in Dulbecco’s Modified Eagle Medium (DMEM, High Glucose) (Invitrogen) supplemented with 10% heat-inactivated FCS, 1% Penicillin/Streptomycin and 0.36% L-Glutamine to obtain DMEM complete. Cell vials were stored in liquid nitrogen and passaged twice before injection. Mycoplasma testing was performed every 2 months by PCR on *in vitro* propagated cultures. No additional authentication method was performed.

### Mouse tumour model and Influenza A virus infection

Mice were injected subcutaneously with B16F1, CT26, or LLC tumour cells in PBS enriched with Matrigel Growth Factor Reduced Basement Membrane Matrix (Corning). Tumour volume was measured using a calliper at indicated times. On day four or ten after tumour cell transplantation, mice were anesthetized by i.p. injection of ketamine (100 mg/kg body weight) and xylazine (5 mg/kg body weight) solution. Mice were then infected intranasally with 25μl of low, non-lethal dose (150 PFU/mL) or medium (300 PFU/mL; corresponds to LD50) dose influenza A/PR/8/34. Virus stock was prepared in embryonated chicken eggs and stored at −80°C. For infection with inactivated virus, 0.05 μg in 50 μL inactivated virus per mouse were applied. Body weight loss was monitored every day.

### Friend Virus (FV) infection

The FV complex contained B-tropic F-MuLV and polycythemia-inducing SFFV. The stock was prepared as described previously [41]. Mice were infected intravenously with 20,000 spleen focus-forming units (SFFU) or 10,000 SFFU for acute infection. The stock was lactate dehydrogenase virus (LDV)-free. Since FV infection leads to splenomegaly [42], the spleen weight was determined to confirm acute FV infection (S3 Fig).

### Influenza virus plaque assay

Viral loads were determined by plaque assay as described previously [43]. Briefly, lungs were obtained from IAV-infected mice 8 dpi and subsequently homogenized in PBS containing 0.3% bovine serum albumin (BSA). Homogenized lungs were centrifuged for 8 min at 6,797 rcf. Lung supernatants were serially diluted in Opti-MEM (Invitrogen) containing 0.3% BSA and added to MDCK cells on a 12-well-plate for 1 h at room temperature. Afterwards, supernatants were discarded and DMEM complete containing 3% Avicel (Sigma Aldrich), trypsin (0.4 μg/mL) and gentamycin (100 μg/mL) was added for 72 h at 37°C. To count the plaques, MDCK cells were fixed for 30 min with 4% paraformaldehyde (PFA). Afterwards, the MDCK cell layer was stained with 0.3% crystal violet (Roth).

### Single cell isolation and flow cytometry analysis

For organ analysis, mice were euthanized at indicated times. Single-cell suspensions from tumours, spleens were minced and filtered through a 100-μm cell strainer. Lungs were perfused with PBS, dissected and incubated in IMDM supplemented with 5% FCS, 0.5 mg/mL DNase I (Roche) and 0.16 mg/mL collagenase for 45 min at 37°C prior to mashing through a 100-μm cell strainer. Mashed organs were treated with erythrocyte lysis buffer and finally, cells were suspended in PBS supplemented with 2% FCS and 2 mM EDTA for staining.

Single cells were incubated with fluorochrome-labeled anti-mouse antibodies. The following monoclonal antibodies were used: αCD4 (clone RM4-5), αCD8 (clone 53–6.7), αThy1.1 (clone OX-7) were obtained from BD Biosciences Pharmingen. αPD-1 (clone 29F.1A12), αIFNγ (clone XMG1.2) and αCD43, directed against its activation-associated glycosylated isoform (clone 1B11) were purchased from BioLegend. αGzmB (clone GB12) antibody was purchased from Invitrogen. αKi67 (clone SolA15), αCXCR3 (clone CXCR3-173) and αFoxP3 (clone FJK-16s) were purchased from eBioscience. αTIM-3 (clone 215008) was purchased from R&D systems. To detect B16 melanoma-specific CD8^+^ T-cells, a phycoerythrin (PE)-conjugated recombinant MHC I H-2Db tetramer specific for gp100 (MBL international) was used. For detection of Influenza virus-specific CD8^+^ T-cells, a PE-conjugated recombinant MHC I H-2Db NP-specific tetramer (MBL international) was used.

Intracellular staining for GzmB, IFNγ, and Ki67 was performed according to the Foxp3 / Transcription Factor Staining Buffer Set (eBioscience) as described previously [44]. For IFNγ staining, cell suspensions were restimulated for 4 h with phorbol myristate acetate and ionomycin in the presence of brefeldin A before staining. In all stainings, a fixable viability dye (eBioscience) was used to exclude dead cells from analysis. Data were acquired using an LSR II instrument using DIVA software (BD Biosciences Pharmingen).

Viable cells were counted in each sample to calculate absolute cell numbers. Generally, at least 2 × 10^6^ cells per sample were analysed in flow cytometric analysis.

### In vitro cytotoxicity assay

CD8^+^ T-cells were isolated from tumours of IAV infected or uninfected mice 12 days after tumour inoculation with the CD8^+^ tumour-infiltrating lymphocyte (TIL) isolation kit (Miltenyi Biotec). 1x10^5^ CD8^+^ T-cells were co-cultured with 1x10^4^ eFluor670 (eBioscience) labelled B16 tumour cells. After 18 h B16 cells were detached using trypsin and stained with fixable viability dye (eBioscience). Cytotoxic capacity was calculated by normalizing the frequency of viable B16 cells after co-culture with tumour-derived CD8^+^ T-cells to viable B16 cells without CD8^+^ T-cells.

### Organ biopsies for cytokine detection

From tumours or lungs, biopsies of 20–60 mg weight were separated. Biopsies were bead-beaten two times at 4 m/s for 20 s in a FastPrep-24 5G homogenizer (MP Biomedicals) in 500 μL medium. Homogenates were centrifuged at 6,797 rcf for 8 min at 4°C and supernatants were stored at -80°C until further use.

### Cytokine analysis

Cytokine/chemokine concentrations of IFNγ, IL-12, TNFα and CXCL10 in the supernatants of tumour or lung biopsies were measured *via* Luminex analysis (R&D Systems) using a Luminex MAGPIX instrument. For analysis, levels were normalised to the respective organ weights. RNA expression of *Cxcl9* and *Cxcl11* in the tumour was analysed by RT-qPCR.

### RT-qPCR

Total RNA was isolated from homogenized lung or tumour biopsies using the RNeasy Fibrous Tissue Mini Kit (Qiagen) according to the manufacturer’s recommendations. RNA concentration was determined with NanopDrop 1000 Spectrophotometer (NanoDrop). Reverse transcription of RNA was performed using M-MLV reverse transcriptase (Promega) and OligodT mixed with Random Hexamer primers (Invitrogen) and cDNA synthesis was validated by a PCR on ribosomal protein S9 (RPS9). Quantitative real-time PCR analysis was performed using the Maxima SYBR Green Master Mix (Thermo Fisher Scientific) on an ABI PRISM cycler (Applied Biosystems). To quantify the viral load, a conserved sequence in the matrix gene M1 of influenza A virus was measured using specific primers (5’-CTTCTAACCGAGGTCGAAACG-3’; 5’-AGGGCATTTTGGACAAAGTCGTCTA-3’) as described previously [43]. Likewise, relative Cxcl9 and Cxcl11 mRNA levels were measured with specific primers (5’-AAGTTAGCCTGTGTGGGAGC-3’; 5’-TTACCGAAGGGAGGTGGACA-3’ for Cxcl9 and 5’-AGGAAGGTCACAGCCATAGC-3’; 5’-CGATCTCTGCCATTTTGACG-3’ for Cxcl11). Relative mRNA levels were calculated with included standard curves for each gene and normalization to the housekeeping gene RPS9 (5′-CTGGACGAGGGCAAGATGAAGC-3’; 5′-TGACGTTGGCGGATGAGCACA-3’).

### Inhibitors, blockades and cell depletions

For cell depletion studies, mice were treated i.p. with anti-mouse CD8α (clone YTS169.4, BioXCell) for CD8 depletion, anti-mouse CSF1R (clone AFS98, BioXCell) for macrophage depletion, anti-mouse NK1.1 (clone PK136, BioXCell) for NK cell depletion, or anti-mouse CXCR3 (clone CXCR3-173, BioXCell) for CXCR3 neutralization on days 4, 7 and 10 post tumour transplantation with 200 μg each; or with anti-mouse IFNγ (clone XMG1.2, BioXCell) every other day starting on day 4 post tumour transplantation, with 250 μg initially, followed by 200 μg each. To block IFN I signalling, mice were injected i.p. with 250 μg anti-mouse IFNα receptor I (IFNAR)-1 blocking antibody (clone MAR1-5A3, BioXcell) on days 4, 7 and 10 post tumour transplantation. Lymphocyte migration was blocked by i.p. application of 1 mg/kg Fingolimod (FTY720, Santa Cruz Biotechnology) every second day upon infection.

### Transfer of CD8^+^ T-cells

To follow lymphocyte migration, CD8^+^ T-cells were isolated 11 days post transplantation from tumours of Thy1.1^+^-mice *via* magnetic cell separation (MACS) using the mouse CD8 TIL MicroBeads kit (Miltenyi Biotec) on an autoMACS Pro Separator. Isolated CD8^+^ T-cells were tested for purity (~89.4%) *via* flow cytometry. Subsequently 1.5×10^5^ cells per mice were adoptively transferred into infected or non-infected tumour-bearing wildtype mice. 2 days post i.v. transfer, organs of infected and non-infected recipient mice were analysed to detect Thy1.1^+^ CD8^+^ T-cells.

### Transfer of influenza-specific CD8^+^ T-cells

In order to estimate the impact of influenza-specific CD8^+^ T-cells on tumour growth control, CD8^+^ T-cells were isolated from the lung of IAV-infected mice 8 dpi by MACS using the mouse CD8α MicroBeads kit (Miltenyi Biotec) on an autoMACS Pro Separator (Mitlenyi Biotec). ~6×10^5^ CD8^+^ T-cells were transferred i.v. per tumour-bearing non-infected recipient mouse (7 days post tumour cell transplantation) or 2×10^5^ CD8^+^ T-cells were transferred i.t. per tumour of non-infected recipient mouse (7 days post tumour cell transplantation).

### Statistics

Statistical analyses were performed using Prism 9.4 software (GraphPad).

## Supporting information

S1 Fig Individual tumour size developments. Tumour growth from each individual mouse based on experiments shown in Fig 1.
(TIF)

S2 Fig IAV infection suppresses tumour growth dose dependently. 1 x 10^5^ B16F1 (B16) tumour cells were transplanted subcutaneously (s.c.) into the right flank per mouse. 4 days after tumour transplantation, mice were infected intranasally with Influenza A/PR/8/34 (IAV). The tumour volume was measured everyday once palpable upon infection. Data from 2 experiments with 3–4 mice per group per experiment. Error bars represent SEM. Statistical tests on tumour growth development were performed as Two-way-ANOVA followed by Tukey’s multiple comparisons test. **** = p<0.0001
(TIF)

S3 Fig FV infection induced splenomegaly. Spleen weights were taken 12 days after tumour cell transplantation and 8 days after infection, respectively. 4 mice per group Error bars represent SEM. Significance was tested in Tukey’s multiple comparisons test. * = p<0.05, ** = p<0.01, *** = p<0.001.
(TIF)

S4 Fig Immune cell depletion during IAV infection in tumour-bearing mice. Tumours were subcutaneously (s.c.) transplanted and mice were infected with 150 PFU/mL influenza A/PR/8/34 (IAV) as described. 200μg of depleting antibodies against NK1.1 (**a**), CSF1R (**b**), CD4 (**c**) or CD8 (**d**) were applied i.p. on days 4, 7 and 10 after tumour cell transplantation, respectively. Depletion was confirmed by flow cytometry. Representative dot plots of tumours 12 days after tumour cell transplantation are shown.
(TIF)

S5 Fig CD4^+^ T cells are not altered in the tumour upon infection. B16 tumours were transplanted and mice were infected as described in Fig 1. Tumours were analysed by flow cytometry 12 days post transplantation. (**a**) Frequencies of viable CD4^+^ T cells. (**b**) Frequencies of CD43 or Granzyme B (GzmB) of CD43 expressing cells of CD4^+^ T cells. Data from 2 experiments are shown. Error bars represent SEM.
(TIF)

S6 Fig Virus-specific CD8^+^ T-cells infiltrate the tumour but are dispensable for tumour growth suppression. (**a**) B16 tumour-bearing mice were infected with IAV (150 PFU/mL) as described. 8 days post infection mice were sacrificed for flow cytometry analysis of IAV nucleoprotein (NP)-specific CD8^+^ T-cells. Frequencies of CD43, CD43 and Granzyme B (GzmB) or Ki67 expressing cells are shown. (**b**) CD8^+^ T-cells were isolated from IAV-infected lungs 8 dpi and adoptively transferred (ACT) into B16 tumour-bearing recipient mice at indicated time point. Data from 2 experiments with 4 mice per group per experiment. (**c**) CD8^+^ T-cells were isolated from IAV-infected lungs 8 dpi and transferred intra-tumouraly (i.t.) at indicated time point. 4–5 mice per group. Error bars represent SEM. Statistical tests between two groups were performed as unpaired Student’s t-tests. Statistical tests on tumour growth development were performed as Two-way-ANOVA, in Tukey’s multiple comparisons test.
(TIF)

S7 Fig CXCL9 and CXCL11 are not enhanced in the tumour after IAV infection. B16 tumours were transplanted and mice were infected as described in Fig 1. RNA was extracted from tumours and lungs 12 days post tumour transplantation, corresponding with 8 days post infection. mRNA levels of *Cxcl9* (**a**) or *Cxcl11* (**b**) were measured relative to Rps9 levels. n.d. = not detectable. Error bars represent SEM.
(TIF)
