# Supplementary figures and images for: Influenza virus infection enhances tumour-specific CD8+ T-cell immunity, facilitating tumour control
Source: PLoS Pathog. 2024 Jan 25;20(1):e1011982. doi: 10.1371/journal.ppat.1011982 (PMC10846710; doi:10.1371/journal.ppat.1011982)

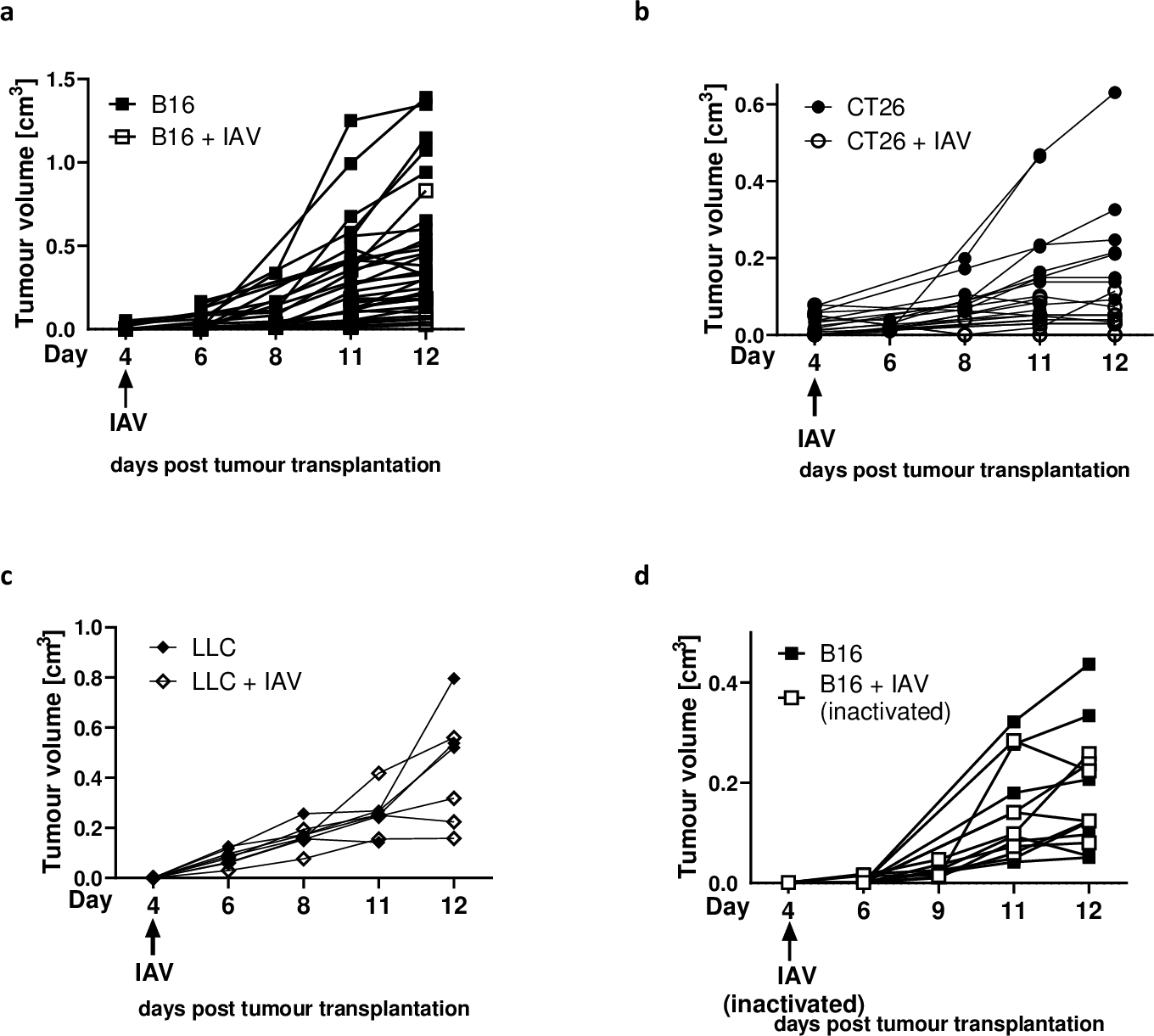

Supplement: S1 Fig — Tumour growth from each individual mouse based on experiments shown in Fig 1. (TIF) [file ppat.1011982.s001.tif]

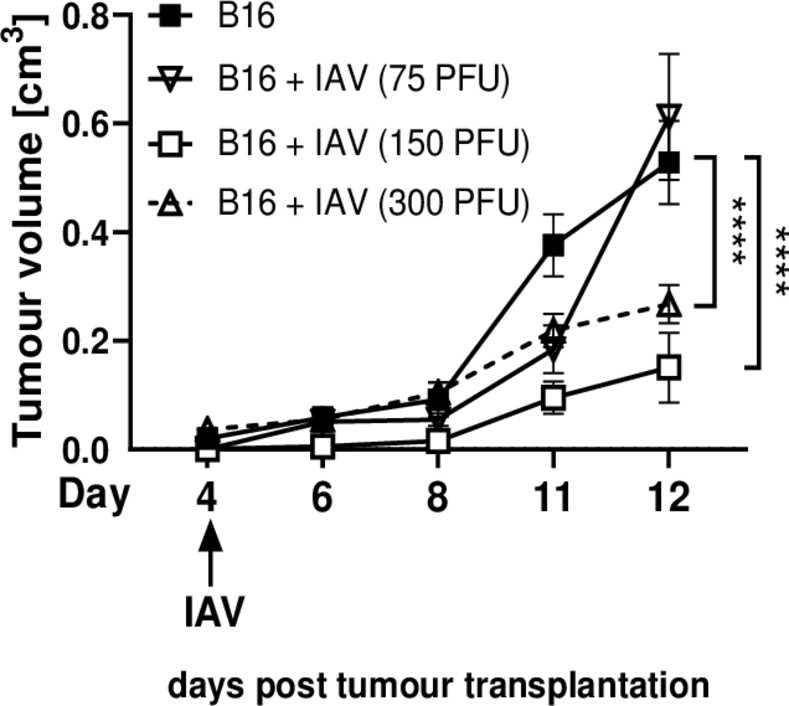

Supplement: S2 Fig — 1 x 10^5^ B16F1 (B16) tumour cells were transplanted subcutaneously (s.c.) into the right flank per mouse. 4 days after tumour transplantation, mice were infected intranasally with Influenza A/PR/8/34 (IAV). The tumour volume was measured everyday once palpable upon infection. Data from 2 experiments with 3–4 mice per group per experiment. Error bars represent SEM. Statistical tests on tumour growth development were performed as Two-way-ANOVA followed by Tukey’s multiple comparisons test. **** = p<0.0001 (TIF) [file ppat.1011982.s002.tif]

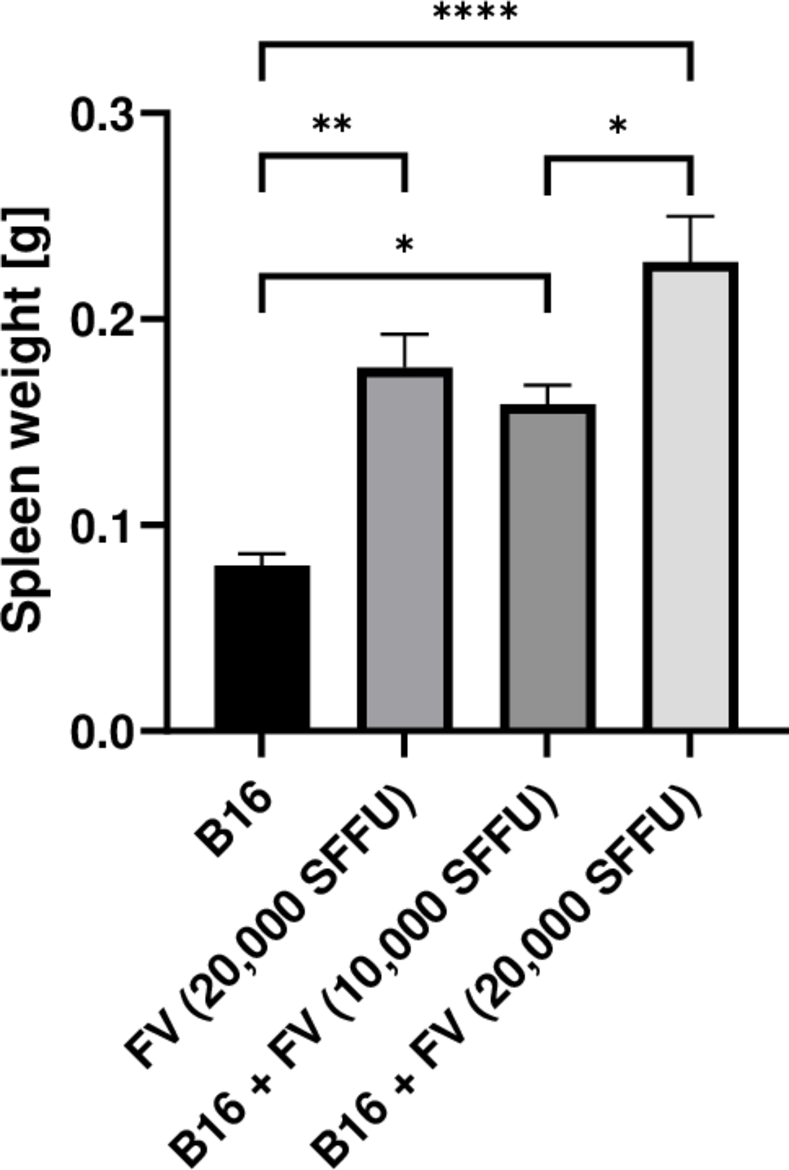

Supplement: S3 Fig — Spleen weights were taken 12 days after tumour cell transplantation and 8 days after infection, respectively. 4 mice per group Error bars represent SEM. Significance was tested in Tukey’s multiple comparisons test. * = p<0.05, ** = p<0.01, *** = p<0.001. (TIF) [file ppat.1011982.s003.tif]

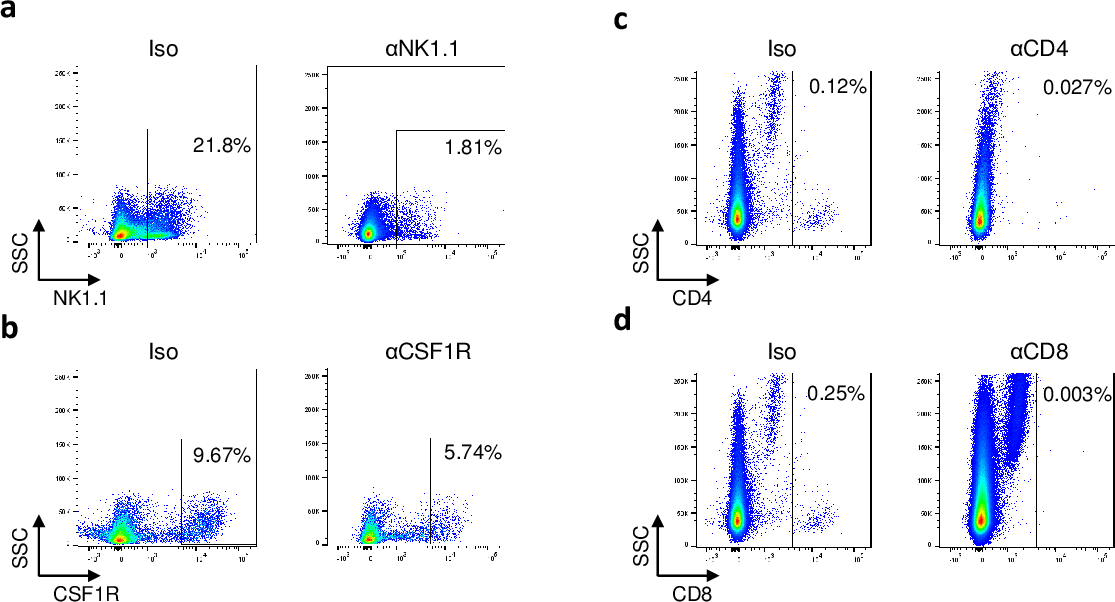

Supplement: S4 Fig — Tumours were subcutaneously (s.c.) transplanted and mice were infected with 150 PFU/mL influenza A/PR/8/34 (IAV) as described. 200μg of depleting antibodies against NK1.1 (a), CSF1R (b), CD4 (c) or CD8 (d) were applied i.p. on days 4, 7 and 10 after tumour cell transplantation, respectively. Depletion was confirmed by flow cytometry. Representative dot plots of tumours 12 days after tumour cell transplantation are shown. (TIF) [file ppat.1011982.s004.tif]

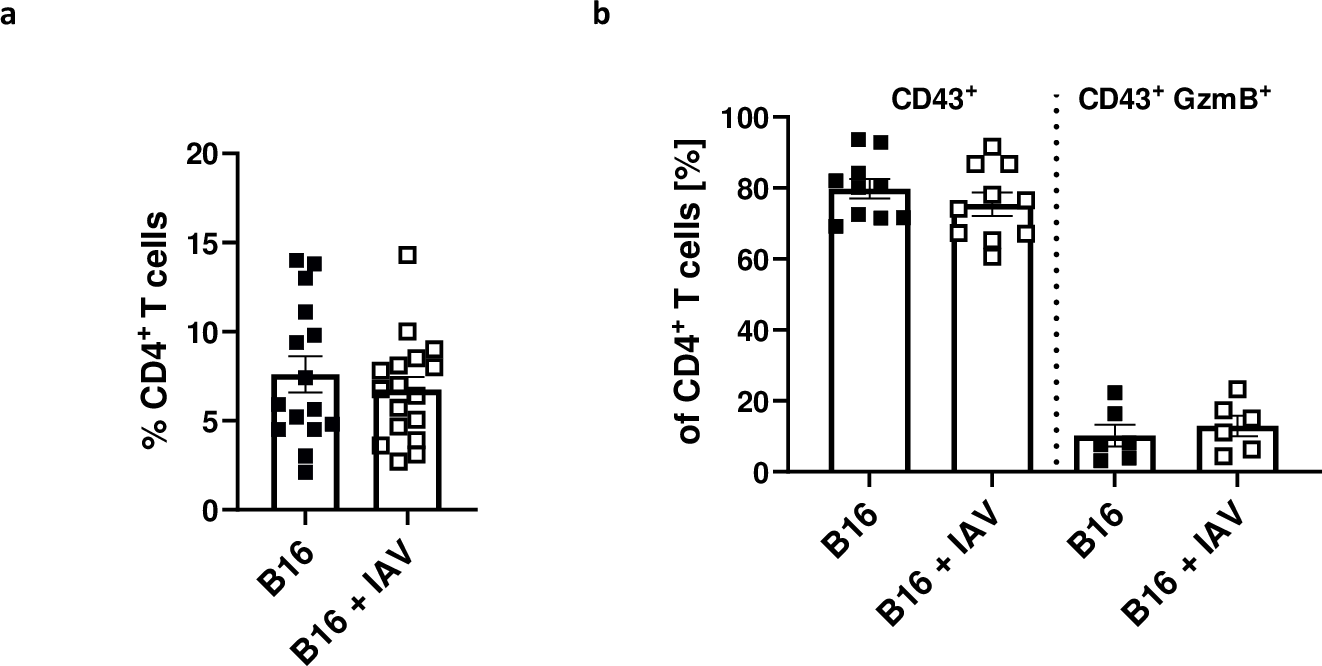

Supplement: S5 Fig — B16 tumours were transplanted and mice were infected as described in Fig 1. Tumours were analysed by flow cytometry 12 days post transplantation. (a) Frequencies of viable CD4+ T cells. (b) Frequencies of CD43 or Granzyme B (GzmB) of CD43 expressing cells of CD4+ T cells. Data from 2 experiments are shown. Error bars represent SEM. (TIF) [file ppat.1011982.s005.tif]

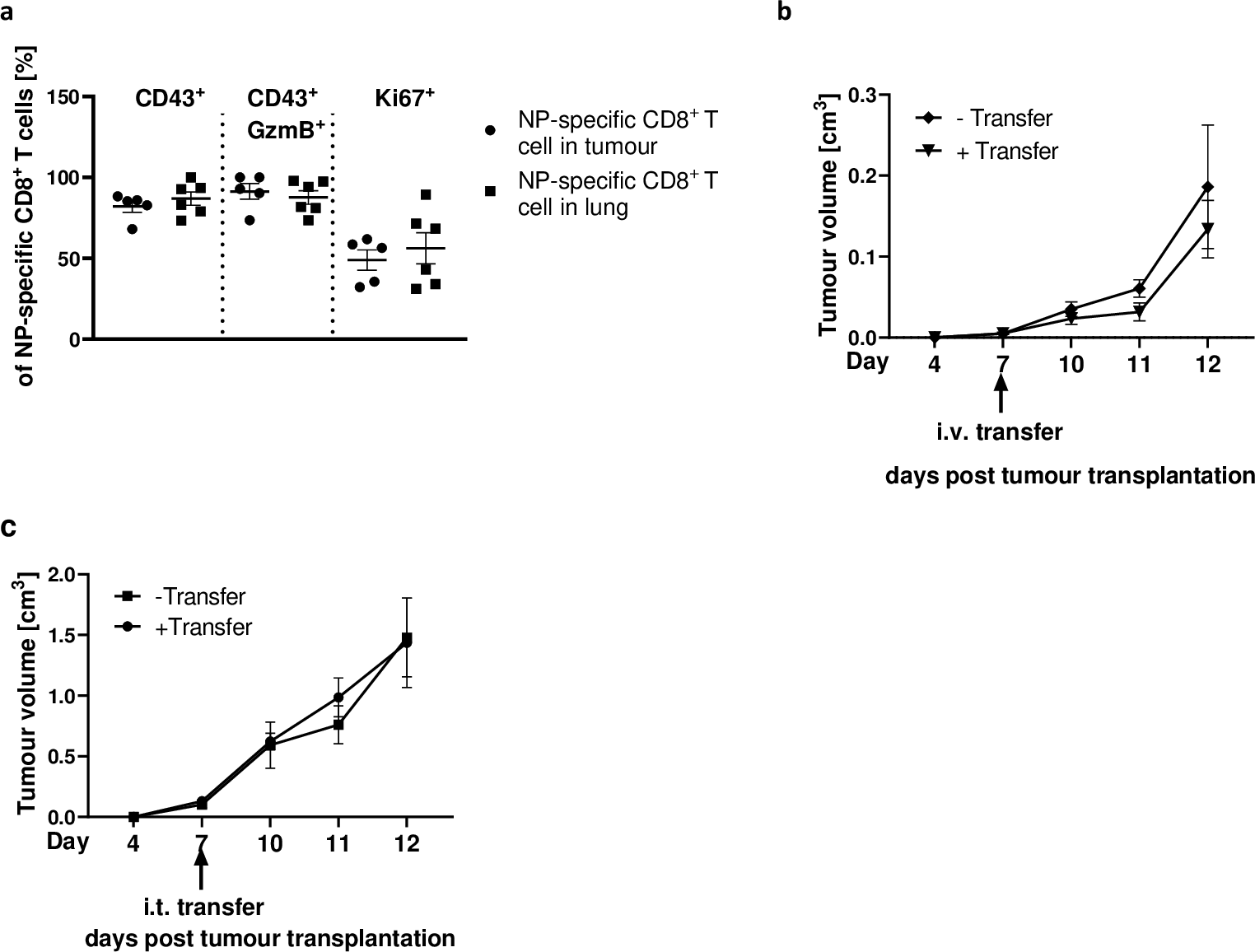

Supplement: S6 Fig — (a) B16 tumour-bearing mice were infected with IAV (150 PFU/mL) as described. 8 days post infection mice were sacrificed for flow cytometry analysis of IAV nucleoprotein (NP)-specific CD8+ T-cells. Frequencies of CD43, CD43 and Granzyme B (GzmB) or Ki67 expressing cells are shown. (b) CD8+ T-cells were isolated from IAV-infected lungs 8 dpi and adoptively transferred (ACT) into B16 tumour-bearing recipient mice at indicated time point. Data from 2 experiments with 4 mice per group per experiment. (c) CD8+ T-cells were isolated from IAV-infected lungs 8 dpi and transferred intra-tumouraly (i.t.) at indicated time point. 4–5 mice per group. Error bars represent SEM. Statistical tests between two groups were performed as unpaired Student’s t-tests. Statistical tests on tumour growth development were performed as Two-way-ANOVA, in Tukey’s multiple comparisons test. (TIF) [file ppat.1011982.s006.tif]

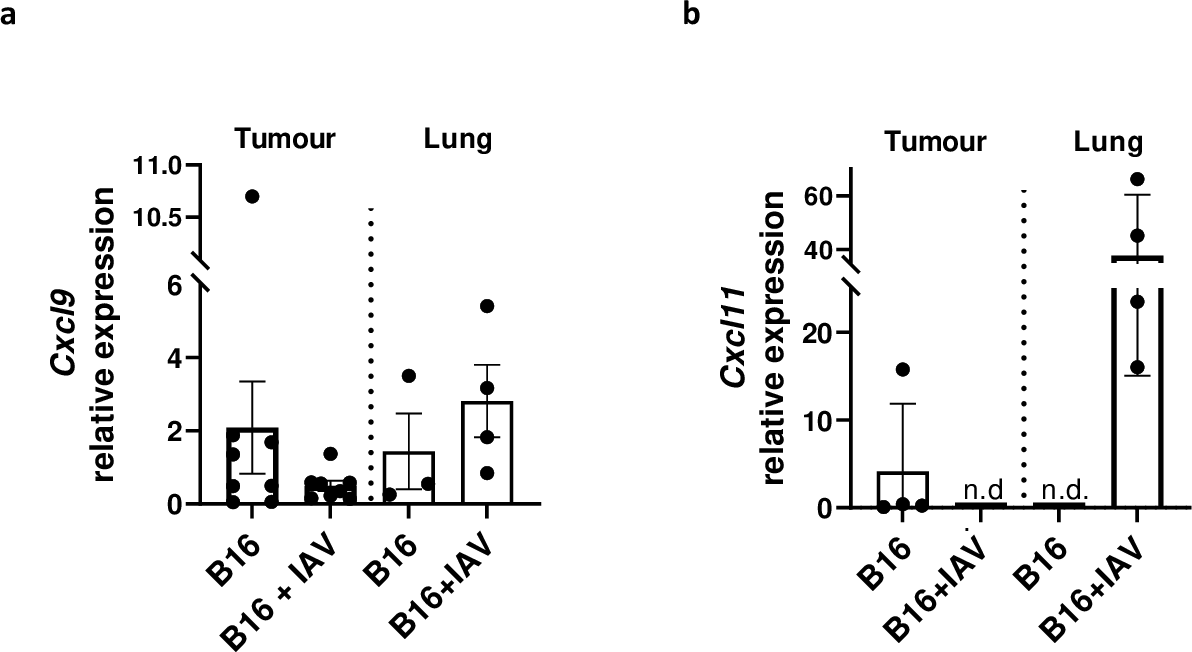

Supplement: S7 Fig — B16 tumours were transplanted and mice were infected as described in Fig 1. RNA was extracted from tumours and lungs 12 days post tumour transplantation, corresponding with 8 days post infection. mRNA levels of Cxcl9 (a) or Cxcl11 (b) were measured relative to Rps9 levels. n.d. = not detectable. Error bars represent SEM. (TIF) [file ppat.1011982.s007.tif]
